# Finding your feet: The development of a podiatry intervention to reduce falls in care home residents

**DOI:** 10.1186/1757-1146-8-S1-A7

**Published:** 2015-04-20

**Authors:** Gavin Wylie, Zoë Young, Roberta Littleford, Frank Sullivan, Joanne Coyle, Brian Williams, Hylton Menz, Simon Ogston, Jacqui Morris

**Affiliations:** 1 Social Dimensions of Health Institute, Dundee University, UK; 2 Department of Podiatry, NHS Tayside, Dundee, UK; 3 Department of Podiatry, Allied Health Professions Directorate, NHS Tayside, Dundee UK; 4 Tayside Medical Science Centre, Ninewells Hospital and Medical School, Dundee, UK; 5 Department of Family & Community Medicine, University of Toronto, North York General Hospital, Canada; 6 Social Dimensions of Health Institute, Dundee University, Dundee, UK; 7 Nursing Midwifery and Allied Health Professions Research Unit, Stirling University, UK; 8 Lower Extremity and Gait Studies Program, La Trobe University, Australia; 9 Centre for Biomedical Science and Public Health, University of Dundee, UK

## Introduction

People who live in care homes often fall. Foot and ankle muscle weakness, sub-optimal footwear, and common foot problems such as corns and hallux valgus are known and potentially modifiable contributory factors to falls in older people. Conducting a randomised controlled trial in a care home setting to address these issues is challenging and presents a number of uncertainties that need to be addressed prior to undertaking a phase III trial. Therefore, this study used a qualitative approach to assess the feasibility and acceptability of a podiatry falls intervention to care home residents and staff. The data acquired during this qualitative preparatory phase will inform the conduct of a subsequent exploratory randomised controlled trial of a podiatry intervention to reduce falls in care homes.

## Methods

### Participants

Permanent care home residents with a history of falls, mini mental state examination (MMSE) >9, ability to provide informed consent (n=8); staff (n=5).

### Intervention

Residents, supported by care home staff, participated in a 3-month feasibility-testing phase of an intervention (footwear and orthoses provision, toe and ankle muscle strengthening programme).

### Evaluation

Exercise frequency was recorded in logbooks by staff. To assess acceptability and perceptions of feasibility at the conclusion of the 3-month testing period, face to face semi-structured interviews were conducted.

### Data analysis

Descriptive data of exercise frequency were calculated. Analysis of the qualitative data employed a constant-comparative process embedded within the wider framework method to identify emerging themes and concepts to inform the intervention remodelling and development.

## Results

### Fidelity

30/57(52.6%) logbooks returned; 11(19.3%) completed in full. Adherence ranged between exercises not having been completed at all in some weeks, to three times per week (optimal) in others.

### Facilitators

Participation in the programme was well received and fitted into care home routines. The exercise component of the intervention was easily carried out and took no longer then 10 minutes to complete. Participants reported that explanation of the aims of the programme at each exercise session was beneficial to adherence. Some residents saw peer support as important; however other residents preferred one-to-one sessions. Footwear and orthoses were well received by the participants.

### Barriers

Discomfort during exercises, cognitive impairment and illness were barriers reported by residents and staff. A major barrier to adherence was limited access for all staff to training resulting in exercises not being performed when trained staff were not available.

## Conclusions

A podiatry intervention to reduce falls in care homes is feasible and acceptable. Delivery to residents should be tailored to individual preferences (taking into account goals, targets, and information). Accessing training via DVD or an online resource may improve the reach of the training, facilitating adherence and fidelity. These findings have informed intervention development and modes of delivery for an exploratory randomised controlled trial that is currently underway.

## Funding

Chief Scientist Office, Scottish Govenrment, award number CZH/4/701

